# Supplementary material for: Anatomical organization of presubicular head-direction circuits
Source: eLife. 2016 Jun 10;5:e14592. doi: 10.7554/eLife.14592 (PMC4927294; doi:10.7554/eLife.14592)
Supplement: Figure 6—source data 1. — The table summarizes the main electrophysiological properties of L2 and L3 neurons (source data for Figure 6A (C and D). The numbers of neurons and the p values are indicated. All p values are from Mann-Whitney U test, except for ‘% of HD cells’ (Fisher’s exact test). DOI: http://dx.doi.org/10.7554/eLife.14592.013 [file elife-14592-fig6-data1.docx]

**Figure 6 – source data 1**

|  | **L2 Neurons** | **L3 Neurons** | **P value** |
| --- | --- | --- | --- |
| **Spike Half-Width (ms)** | 0.35 ± 0.10 (n=11) | 0.28 ± 0.05 (n=22) | p=0.028 |
| **Spike Negativity Ampl. (ms)** | -0.09 ± 0.13 (n=11) | -0.35 ± 0.16 (n=22) | p<0.001 |
| **Average Firing Rate (Hz)** | 2.5 ± 2.5 Hz (n=11) | 2.4 ± 2.6 Hz (n=25) | p=0.312 |
| **HD Index** | 0.37 ± 0.26 (n=11) | 0.83 ± 0.21 (n=22) | p<0.001 |
| **% of HD Cells** | 0/11 | 18/25 | P<0.001 |
| **Theta Index** | 3.36 ± 2.31 (n=10) | 1.27 ± 0.79 (n=22) | p=0.007 |
| **Bursting Index** | 0.043 ± 0.038 (n=11) | 0.11 ± 0.15 (n=22) | p=0.657 |
